# On the Employment of Magnetic Electricity in Certain Forms of Paralysis

**Published:** 1846-11

**Authors:** J. M. Neligan


*On the Employment of Magnetic Electricity in Certain Forms of Paralysis.* By J. M. Neligan, M.D., &c., &c.—One of the principal reasons why electricity and galvanism have fallen into disuse has been the difficulty of their application, in consequence of the large and inconvenient size of the apparatus; but now that such great improvements have been effected in the construction of magnetico-electrical machines, this objection is removed, yet it is to be feared that its too indiscriminate employment may bring it into disrepute. In the following cases, Dr. Neligan has confined its use to certain forms of paralysis, in which there appeared no organic lesion, but merely a want of nervous energy. He thus describes the apparatus used on these occasions:

The instrument which I have employed is that which consists of a small battery, in connection with a frame, on which is fixed an upright straight magnet, surrounded with a bundle of iron wires, round which are coiled some thousand yards of insulated large and small copper wire, divided into seven different portions, each of which terminates separately in a small brass nob, brought up through the bottom of the frame. The shocks are produced by the continuity of the stream of electricity being broken, by the alternate attraction and repulsion, by the magnet of a piece of soft iron, which is kept in contact with a platinized screw, by means of a piece of watch spring. This instrument possesses many advantages, from its portability, the great readiness with which it may be applied, and the facility with which the shock may be regulated; the latter being effected by taking in one or more additional coils of wire, which is done simply by moving a brass regulator attached to the frame to the different knobs, in which, as it is said above, the coils of insulated wire terminate. The profession in Dublin are supplied by Mr. Robinson, of 65 Grafton street, the cost of the instrument beng from £3 10s. to £4 4s.

The form of paralysis in which I have found magnetic-electricity prove most useful, is when a single muscle, or a certain class of muscles, have become paralyzed from a special or local cause. Thus, I have derived peculiar benefit from its use in that particular form of paralysis of the muscles of the fore-arm, which is produced by the action of lead, and which is so frequent a sequence of painter's colic; as also in those cases where a single muscle becomes paralyzed, either from exposure to a draught of cold air, or from continued pressure on the nerve by which the muscle is supplied. In the treatment of hemiplegia or paraplegia, even in their chronic stage, when, of course, the use of any plan of stimulant treatment could alone be had recourse to, I have not seen any benefit produced by the use of electricity; nor, indeed, in any instance where the cause of the disease could be traced to the brain or spinal marrow. In short, I believe that electricity must be looked upon as a remedial agent applicable to local and not to general paralysis.

The first case was that of a girl nine years of age, who had paralysis of the right sterno-mastoid muscle, resulting from inflammation of the cervical fascia. This took place in November, 1843; the result was wryneck; the right sterno-mastoid was paralysed, and the left having nothing to antagonize, it assumed a state of spastic rigidity producing this deformity. The treatment at first was small doses of saccharated carbonate of iron, with the view of improving the general health, and afterwards to make use of magnetic electricity. In three weeks' time, he says:

I commenced the use of the electro-magnetic machine with the child by applying the conductors of the instrument in which pieces of sponge, moistened with salt and water, were fastened, one to the origin, and the other to the insertion of the right sterno-mastoid muscle. The application was at first continued for only a quarter of an hour, but was gradually prolonged until she could bear it for half an hour at a time, which period I never exceeded. The weakest power of the instrument was used, and it was applied only twice a-week. After the third or fourth application, a decided amendment was visible; the sternal end of the muscle being the first to regain its power, as was evident from its becoming fuller, and contracting more strongly under the shocks. The head gradually assumed its natural position, and was perfectly straight on the 20th of May, at which time, also, not the least development of the muscle of either side could be perceived.

In this case the result of subsequent experience leads me to believe, that the cure would have been much accelerated had the electrical shocks been more frequently applied; but as it was the first case in which I employed the electro-magnetic machine, I was cautious in its application.

The second case in which Dr. Neligan tried it, was one of painters' colic, succeeded by almost complete palsy of the muscles of both fore-arms. After the colicky pains were removed and the bowels opened, he was subjected to the influence of magnetic-electricity; though previously he could not stir his hands, they immediately closed on the conductor. The application was continued for half an hour at a time, and in four weeks he was quite cured. The next was a case of paralysis of the right shoulder, from the effects of damp. He was a sailor, and had slept for nights together in wet clothes on deck; his right arm at first began to feel heavy and numb; it pained him to stir it; and he gradually lost all power over it. Blisters and moxas had been applied without any benefit. Electro-galvanism was at length made use of by Dr. Neligan, on the 20th of December, and by the 30th he could use his arm nearly as well as ever. Dr. Neligan adds:]

From what I have already said, I need scarcely add, that I have employed magnetic electricity in a great number of cases of hemiplegia and paraplegia, in their chronic stage; but my experience leads me to place little reliance on its application in such cases. Indeed, so far from proving beneficial, I have seen it, in some apoplectic individuals, prove absolutely injurious by its effects in hurrying the circulation. If the above short observations and cases lead to a more correct appreciation of a useful therapeutic agent—hitherto too much overlooked by the regular practitioner—my object in making them public will be completely answered.—*Monthly Jour. of Med. Science.*

